# Evaluation of the safety, efficacy, effectiveness and cost-effectiveness of implantable Holter for prolonged monitoring in patients with previous stroke: a systematic review

**DOI:** 10.3205/hta000137

**Published:** 2023-09-26

**Authors:** Carmen Martín-Gómez, Elena Baños-Álvarez, Rebeca Isabel-Gómez, Juan Antonio Blasco-Amaro

**Affiliations:** 1 Área de Evaluación de Tecnologías Sanitarias de Andalucía (AETSA), Fundación Pública Andaluza Progreso y Salud, Seville, Spain; 2 Servicio de Salud Pública. Distrito Sanitario de Atención Primaria, Seville, Spain

**Keywords:** atrial fibrillation, stroke, implantable Holter monitor, diagnosis, systematic review

## Abstract

**Background:**

Atrial fibrillation (AF), which is associated with cryptogenic stroke, is the most common sustained arrhythmia in the general population. Because AF is asymptomatic and intermittent, its detection rate increases with the duration of monitoring. The objective of this study is to review the available evidence on the safety, efficacy, effectiveness and cost-effectiveness of AF diagnosis by prolonged monitoring with an implantable Holter monitor in adult patients with idiopathic or cryptogenic stroke of suspected cardioembolic origin, compared to conventional monitoring.

**Methods:**

Two independent reviewers performed a systematic review of the literature, identifying relevant studies through a structured search of Medline (Ovid), EMBASE, Web of Science and Cochrane Library and the databases of national and international health technology assessment agencies. The quality of the included studies was assessed with AGREE-II, AMSTAR-2 and CHEC. GRADE criteria were used to summarise the evidence

**Results:**

Four of the 211 papers identified were included: 1 clinical practice guideline, 2 systematic reviews, and 1 economic evaluation. The quality of the evidence reviewed was low. An implantable Holter monitor might be more effective in detecting AF than conventional monitoring. Serious adverse events were similar in both groups. The economic evaluation suggests that the technology is cost-effective.

**Conclusions:**

The available evidence suggests the diagnostic superiority of the implantable Holter monitor over the traditional Holter monitor. Due to the low quality of the evidence, further and higher quality studies on these technologies are needed before solid conclusions can be drawn.

## Introduction

 Atrial fibrillation (AF) is the most common type of sustained arrhythmia in the general population, being considered a global epidemic [1]. Its incidence and prevalence have increased in the past 20 years, and is expected to continue to do so over the coming 30 years [2]. In Spain, the OFRECE study found a 4.4% prevalence of AF in both men and women aged over 40 years, a percentage that increased after 60 years [3]. Estimates suggest that by 2060, 17.9 million people in Europe will present this disease [4], making it a major epidemic and public health challenge, particularly in countries with a middle sociodemographic index [2].

Undetected AF may be responsible for cryptogenic stroke, since AF is suspected in 25% to 30% of these cases [5]. AF has traditionally been diagnosed by recording cardiac activity with a conventional 24-hour Holter [6]. Diagnosis is usually straightforward when AF is persistent; however, AF is often asymptomatic and intermittent (paroxysmal). In these cases, the likelihood of detection increases with the duration of monitoring, and they are therefore difficult to detect with traditional Holters [7], [8], [9]. An alternative diagnostic technique is the implantable Holter monitor, a subcutaneous device that provides long-term continuous heart monitoring. In patients with cryptogenic stroke, this device reduces the delay between detection and recording of AF and review of the ECG readout by a specialist. Four implantable Holter devices are currently available: BioMonitor 2 AF, BioMonitor III, Confirm RX and Reveal LINQ. All are authorised by the US Food and Drug Administration [10], [11], [12], [13] and are CE-marked for distribution in Europe [14], [15], [16], [17].

These implantable [11] devices may help clinicians identify AF in patients with cryptogenic stroke. Therefore, aim of this study is to review the state of the evidence on the safety, efficacy, effectiveness and cost-effectivenes of AF diagnosis by long-term monitoring with an implantable Holter monitor in adult patients with idiopathic or cryptogenic stroke of suspected cardioembolic origin compared to diagnosis with conventional monitoring.

## Methods

We performed a systematic literature review. The review protocol was registered in the PROSPERO database (ID: 218809) prior to the start of the study. We used GRADE criteria to summarise the evidence [18], and reported our findings in accordance with the PRISMA (Preferred Reporting Items for Systematic Reviews and Meta-Analyses) statement [19].

### Databases and search strategy

In March 2021, we performed a structured literature search in Medline (Ovid), EMBASE, Web of Science and the Cochrane Library. The search strategy used in each database is described in Attachment 1.

The strategy was supplemented with a manual search of the websites of the main national and international health technology assessment agencies (see Attachment 2). 

Finally, we performed a manual review of the papers referenced in the articles selected for final analysis in our literature review.

#### Study inclusion and exclusion criteria 

The inclusion and exclusion criteria were defined following the PICOS framework (participant, intervention, comparison, outcomes, study design), and are shown in Table 1 (Tab. 1). 

## Results

### Search results

**Figure 1:**
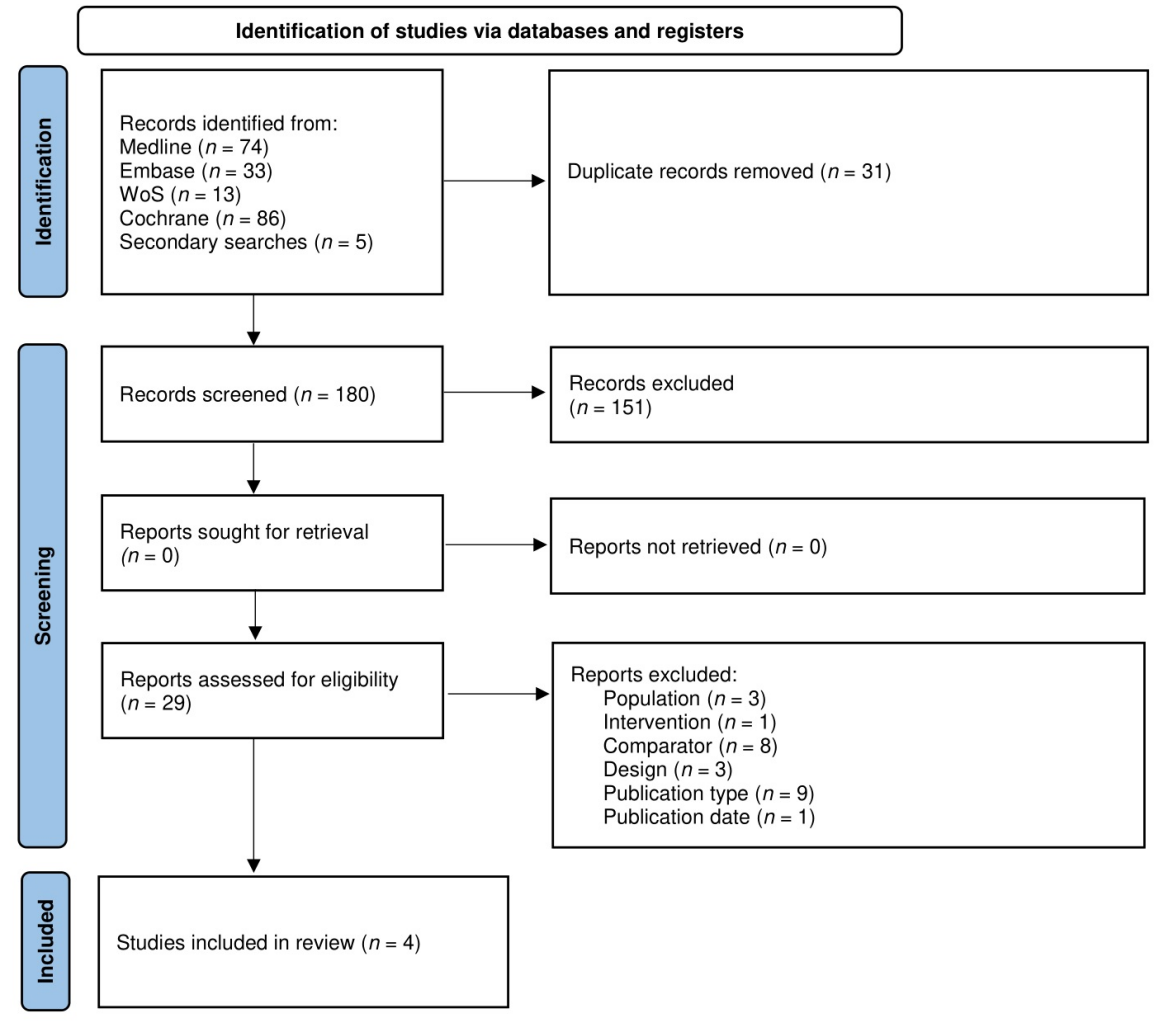
Flow diagram of included studies

After removing duplicates, 180 documents were identified out of a total of 211 papers retrieved in the searches described above. One hundred and fifty-one were excluded after reviewing the title and abstract. Four of the 29 documents that were read in full met the inclusion criteria and were selected for analysis. Figure 1 (Fig. 1) shows the study selection flowchart. The studies excluded after full examination of the text, together with the reasons for exclusion, are listed in Attachment 3. 

#### Quality of included studies

Both systematic reviews [20], [21] were of low overall quality. The clinical practice guideline [22] obtained favourable results in most of the domains. The results obtained for each of the domains were as follows: “scope and purpose”, “clarity of presentation” and “editorial independence” were rated 100%, “stakeholder involvement” and “rigour of development” were rated 72%, and “applicability” was rated 25%. It is important to note that the quality assessment tool used analyses the methodological quality of the guidelines themselves, but not that of the systematic review [23] on which it is based (which in this case is low). The quality of the economic evaluation study [24] was moderate. The assessment of the methodological quality of each document can be found in Attachment 4.

##### Characteristics of the included studies

The studies included were 1 clinical practice guideline [22], 1 economic evaluation [24] and 2 systematic reviews [20], [21]. The economic evaluation [24] was based on a cost-effectiveness Markov model, and the systematic reviews analysed the feasibility of Holter monitors: one [21] analysed cost-utility studies, and the other also analysed the accuracy of the diagnostic tests and the clinical efficacy of the devices [20]. The clinical practice guideline [22] was based on the latter systematic review [20], and included and analysed 1 randomized controlled trial (RCT) and 26 observational studies.

Table 2 (Tab. 2) describes the characteristics of the studies, and Table 3 (Tab. 3) describes the population and target intervention analysed in the economic evaluation and systematic reviews included in this study.

#### Safety 

##### Adverse Events

In the RCT, the rate of non-serious adverse events was higher in the group with the implanted device (18.6%) vs. the group undergoing traditional monitoring (4.1%); the rate of serious adverse events was similar in both groups (25%, 30%) [20]. No device insertion site complications were reported, there were no adverse events during the procedure, and no anticoagulation-related complications [21]. Regarding the observational studies, 5 reported adverse events and another 3 reported no device insertion-related complications without providing more detailed information [22].

##### Removal of the device

In the RCT, 5 (2.4%) devices had to be removed 36 months after implantation due to pocket infection or erosion [20]. Three of the observational studies on the Reveal device (LINQ or XT) reported removals without detailing the reason; 2 others reported premature removals due to skin reactions, migration, or discomfort (0.9% 5.7%), in line with the RCT (2.4%) [20]. At 12-month follow-up, 3.4% of devices were removed in the RCT, in contrast to another study on Reveal XT in which removal was suggested after detection of AF and for other reasons, with 30% patients agreeing to removal of the device during the study (median follow-up 13 months) [20]. 

#### Efficacy and effectiveness

##### Sensitivity and specificity

Although the RCT did not provide data on sensitivity or specificity, it did report that the alerts generated by the devices had to be confirmed by clinical personnel before starting anticoagulation therapy. None of the observational studies compared the diagnostic accuracy of the implantable device to conventional monitoring. However, in 2 of them [25], [26] detection of AF was modelled on data from the included RCT (Reveal XT; n=221) and data from a patient registry (Reveal Linq; n=1,247), using repeated iterations (10,000). Based on the assumption that the implanted device has 100% sensitivity, the goal was to estimate the number of patients in whom AF was not detected using an intermittent follow-up strategy. The results showed that even the best intermittent follow-up strategy detected less than a third of AFs detected by an implanted device [20], [21], [22]. 

##### Diagnostic yield

In the RCT, detection of AF was higher in patients with the implanted device vs. those undergoing conventional monitoring, with AF detected in 8.6% of patients at 6 months and 19% at 36 months in the arm with the implanted device vs. 1.4% of patients at 6 months and 2.3% at 36 months in the conventional monitoring group. In the observational studies, detection of AF was highly variable, ranging from 6.7% to 40.9% in the first reported follow-up period, which in turn ranged from 6 to 24 months. 

##### Time to diagnosis

In the 36-month follow-up described in the RCT, 42 patients in the implanted device group were diagnosed with AF vs. 5 in the group that underwent conventional monitoring. Differences between the monitoring methods used prevent us from drawing conclusions about the average time to diagnosis of AF; what the data do show, however, is that the number of patients diagnosed with AF increased with the duration of monitoring, and this increase was always greater in the implanted device group. Eighteen of the observational studies reported the time to diagnosis of AF, which varied from 7 to 20 months, with median time to first detection of AF ranging from 21 to 217 days.

##### Positive and negative predictive values

Evidence suggests that the positive and negative predictive values for the detection of AF with implantable Holter monitors depends on the patient population, the incidence of AF, the duration of monitoring, and the type of AF [20]. Similarly, estimated negative predictive values ranged from 82.3% to 89.7% in 2 of the observational studies [20]. 

##### Likelihood ratios

Likelihood ratios were not reported in any of the included studies. 

#### Other factors analysed 

##### Holter implantation by clinical staff

Both clinical experts from the evidence assessment group and the companies that market the devices indicate that the most recent Holter models are easy to implant, even by non-physicians, provided the procedure is performed by trained personnel [20].

##### Use of anticoagulants

In the RCT, 90% of patients diagnosed with AF in the implanted device group were subsequently treated with oral anticoagulants; anticoagulation therapy in patients undergoing conventional monitoring was not reported [20]. In 7 of the observational studies, between 90% to 100% of patients diagnosed with AF received anticoagulants, and another study reported the total number of patients treated with oral anticoagulants (n=19) without indicating whether these patients belonged to the group diagnosed with AF or not [20]. 

##### Detection of other arrhythmias

Three of the observational studies indicated that other types of arrhythmia were detected in around 10% of patients with implanted Holter monitors, mainly bradycardia, bigeminy or pauses [20]. 

##### Stroke or transient ischaemic attack (TIA) during follow-up

In the RCT, the data showed a trend towards fewer cases of de novo stroke or TIA in the implanted device arm compared to conventional monitoring [20]. Six of the observational studies provided information on stroke during follow-up, reporting that incidence ranged from 0% to 14.6% one year after Holter implantation. Incidence was higher in patients without detected AF, although it was unclear how many strokes or TIAs in patients with AF occurred before detection of AF [20]. 

#### Cost-effectiveness

All the papers included in this review conclude that the use of implantable Holter could be cost-effective [20], [21], [22], [24]. Specifically, based on the assumption that all the Holter devices evaluated are clinically similar (Reveal Linq, BioMonitor 2-AF and Confirm Rx, and their earlier versions), these devices could be cost-effective at a £20,0000 to £30,000 threshold compared with standard limited-duration monitoring [20], [21] [22]. Furthermore, although long-term monitoring may be economically attractive, incremental cost-effectiveness ratios varied when the traditionally accepted willingness-to-pay threshold was applied [21]. Nevertheless, an implantable Holter was only recommended when AF could not be detected using a conventional device but suspicion persisted [20], [22], [24].

## Discussion

The results of this review show that AF detection is superior in patients with implantable Holter monitors than in patients undergoing conventional monitoring. Nevertheless, in the studies reviewed an implantable Holter is only recommended when conventional monitoring has failed to detect AF in the target population. Serious adverse effects were similar in both implantable and conventional monitoring devices, which may be relevant for patients. The implantable Holter is a cost-effective alternative.

However, our findings must be viewed with caution, mainly due to the limited amount of information available for review: 1 clinical practice guideline [22], 1 economic assessment [24] and 2 systematic reviews [20], [21]. Because of the heterogeneity of the included studies, we were unable to pool the data and draw strong conclusions. The AGREE-II [23], AMSTAR-2 [27] and CHEC [28] assessment tools showed that the overall methodological quality of the studies included was low. For these reasons, the diagnostic effectiveness of implantable Holter monitoring needs to be supported by further, more reliable evidence.

We also suggest that further studies are needed to bridge the knowledge gaps identified in this review, including:

1. studies evaluating the BioMonitor III, the latest device to become available;
2. clinical trials, ideally RCTs, in patients with stroke to compare the clinical utility of the different implantable Holter models (Reveal LINQ, BioMonitor 2-AF, BioMonitor III and Confirm Rx);
3. diagnostic accuracy studies of the results obtained with each implantable Holter monitor model, using 24-hour electrocardiogram monitoring as a consistent reference standard in patients with stroke;
4. studies analysing the risks and benefits of long-term anticoagulation as a secondary prevention strategy in stroke patients with AF, in order to confirm the clinical benefit of using the implantable device to detect additional cases of AF in stroke patients;
5. studies to identify which patients might benefit most from implantable Holter devices (for example, patients that have had more severe strokes or those with risk factors such as diabetes or sleep apnoea).

Finally, underlying assumptions in economic analysis models, for example, annual stroke ratios, affect the interpretation of cost-utility results. These assumptions, therefore, need to be better clarified in order to guide healthcare professionals, healthcare authorities, and hospital managers in their decision-making processes.

Considering the positive impact of implantable Holter monitoring on secondary prevention and in reducing the incidence of stroke, we can expect this technology to have a considerable impact on public health, provided future scientific evidence continues to support its diagnostic effectiveness.

## Conclusions

The evidence from the studies reviewed suggests that identifying AF in patients with cryptogenic stroke using an implantable Holter device, is cost-effective and seems to be superior to conventional monitoring. Further evidence from methodologically sound studies is still needed, specifically, RCTs evaluating all Holter models. It would also be interesting to perform studies comparing the clinical efficacy of the different implantable Holters available. 

## Notes

### List of abbreviations 

**AF:** Atrial fibrillation

**PICOS:** participant, intervention, comparison, outcomes, study design

**PRISMA:** preferred reporting items for systematic reviews and meta-analyses

**RCT:** randomized controlled trial

**TIA:** transient ischaemic attack

### Competing interests

The authors declare that they have no competing interests.

### Acknowledgments

This study was funded by the Spanish Ministry of Health for the development of the Annual Working Plan of the Spanish National Health System’s Network of Health Technology and Performance Assessment Agencies. 

We would like to thank Ricardo Ruiz Granel, from the Arrhythmia Unit of the Hospital Clínico Universitario de Valencia, for reviewing the manuscript. 

### Ethics approval and consent to participate

Not applicable.

### Consent for publication

Not applicable.

### Authors’ contributions

CMG, EBA and JABA planned and designed the research. RIG was responsible for the documentation. CMG and EBA collected and analysed the data and presented the results. The manuscript was drafted by CMG and critically reviewed by EBA, RIG and JABA. CMG, EBA, RIG and JABA approved the final version of the manuscript and are accountable for the content.

## Supplementary Material

Search strategy

Health technology assessment websites which were searched manually

Studies excluded after reading the full text

Methodological quality of the included studies

## Figures and Tables

**Table 1:**
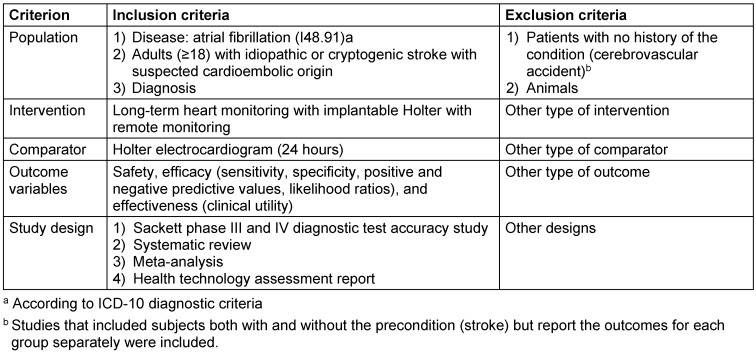
Study inclusion and exclusion criteria

**Table 2:**
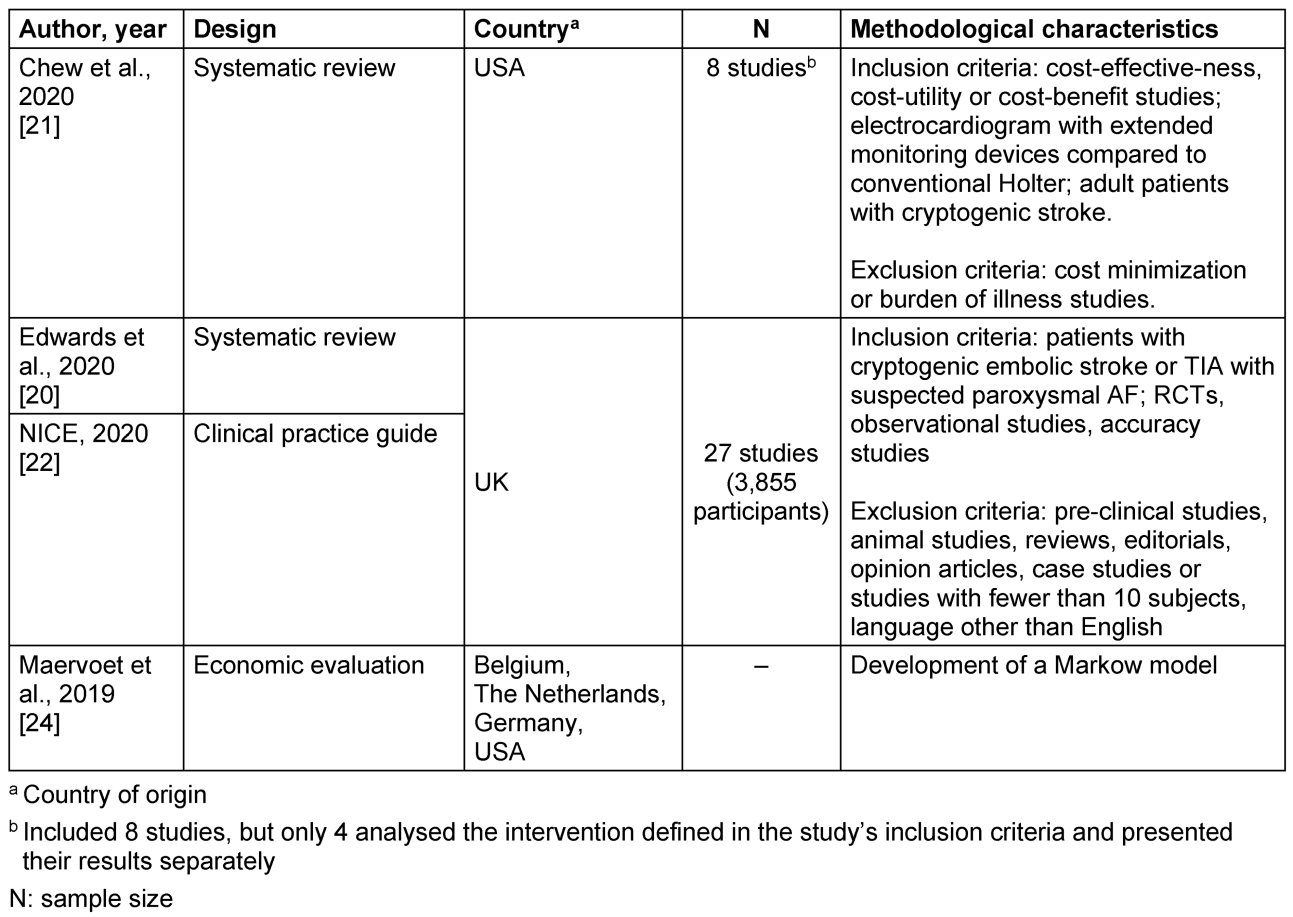
Characteristics of the included studies

**Table 3:**
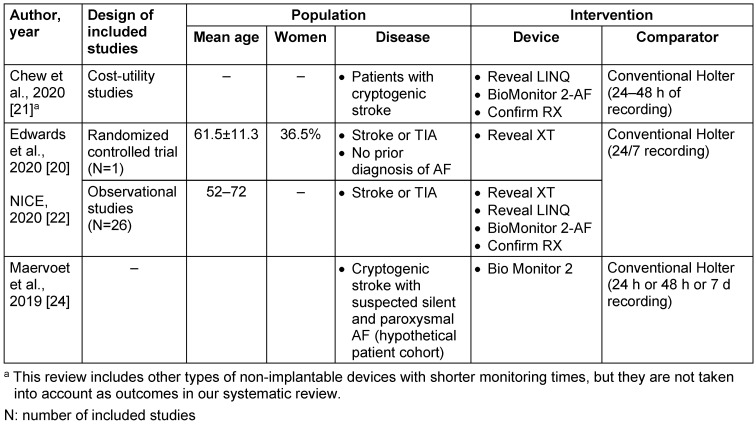
Population and target intervention analysed in the included studies
