# Supplementary material for: Evaluation of the safety, efficacy, effectiveness and cost-effectiveness of implantable Holter for prolonged monitoring in patients with previous stroke: a systematic review
Source: GMS Health Innov Technol. 2023 Sep 26;17:Doc01. doi: 10.3205/hta000137 (PMC10587481; doi:10.3205/hta000137)
Supplement: Search strategy [file HINT-17-01-s-001.pdf]

Search strategy

Medline (Ovid)

Searched on 26 February 2021

Database: Ovid MEDLINE(R) and Epub Ahead of Print, In-Process & Other Non-Indexed Citations, Daily and Versions(R) <2018 to Febrary 26, 2021>
Search Strategy:
----
1 stroke/ or stroke,lacunar/ or brain ischemia/ or brain infarction/ or brain stem infarctions/ or cerebral infarction/ or infarction, middle cerebral artery/ or infarction, posterior cerebral artery/ or hypoxia-ischemia, brain/ or ischemic attack, transient/ or infarction, anterior cerebral artery/
2 (stroke* or ictus or CVA or TIA or apoplex*).ti,ab.
3 ((brain? or cerebr* or cerebell* or intracerebral or intracran* or cerebrovasc*) adj3 (accident* or attack* or infarct* or ischaemic* or ischemic*)).ti,ab.
4 ((cerebro adj vasc*) or (brain adj2 vasc*)).ti,ab.
5 or/1-4
6 ((implant* or insert*) adj2 cardiac adj2 monitor*).mp.
7 ((implant* or insert*) adj3 recorder*).mp.
8 (reveal adj2 (linq* or XT*)).af.
9 biomonitor*.af.
10 (confirm adj2 RX).af.
11 (IMC or IMCs).mp.
12 or/6-11
13 5 and 12
14 limit 13 to yr="2018 -Current"

Embase

Searched on 4 March 2021

#1. 'cerebrovascular accident'/exp OR 'brain ischemia'/exp OR 'brain infarction'/exp OR 'cerebral artery disease'/exp OR 'hypoxic ischemic encephalopathy'/exp
#2. stroke*:ti,ab OR ictus:ti,ab OR cva:ti,ab OR tia:ti,ab OR apoplex*:ti,ab
#3. ((brain* OR cerebr* OR cerebell* OR intracerebral OR intracran* OR cerebrovasc*) NEAR/3 (accident* OR attack* OR infarct* OR ischaemic* OR ischemic*)):ti,ab
#4. cerebro:ti,ab AND near:ti,ab AND vasc*:ti,ab OR ((brain NEAR/2 vasc*):ti,ab)
#5. #1 OR #2 OR #3 OR #4
#6. 'implantable cardiac monitor'/exp
#7. (((implant* OR insert*) NEAR/2 cardiac):ti,ab,kw) AND monitor*:ti,ab,kw
#8. ((implant* OR insert*) NEAR/3 recorder*):ti,ab,kw
#9. (reveal NEAR/2 (linq OR xt)):ti,ab,kw
#10. biomonitor*:ti,ab,kw
#11. (confirm* NEAR/2 rx):ti,ab,kw
#12. imc:ti,ab,kw OR imcs:ti,ab,kw
#13. #6 OR #7 OR #8 OR #9 OR #10 OR #11 OR #12
#14. #5 AND #13
#15. #14 AND (2018:py OR 2019:py OR 2020:py)
#16. #14 AND (2018:py OR 2019:py OR 2020:py) AND [embase]/lim NOT ([embase]/lim AND [medline]/lim) NOT ('conference abstract'/it OR 'conference review'/it)

Available from: Martín-Gómez C, Baños-Álvarez E, Isabel-Gómez R, Blasco-Amaro J. Evaluation of the safety, efficacy, effectiveness and cost-effectiveness of implantable Holter for prolonged monitoring in patients with previous stroke: a systematic review. GMS Health Innov Technol. 2023;17:Doc05. DOI: 10.3205/hta000137

Web of Science
Searched on 4 March 2021

#1 TS=(stroke* or ictus or CVA or TIA or apoplex*)
#2 TS=((brain* or cerebr* or cerebell* or intracerebral or intracran* or cerebrovasc*) NEAR/3 (accident* or attack* or infarct* or ischaemic* or ischemic*))
#3 TS=((cerebro NEAR/1 vasc*) or (brain NEAR/2 vasc*))
#4 #3 OR #2 OR #1
#5 TS=(((implant* or insert*) NEAR/2 cardiac) NEAR/2 monitor*)
#6 TS=((implant* or insert*) NEAR/3 recorder*)
#7 TS=(reveal NEAR/2 (linq* or "XT"))
#8 TS=(biomonitor*)
#9 TS= (confirm NEAR/2 "RX")
#10 TS=(IMC or IMCs)
#11 #10 OR #9 OR #8 OR #7 OR #6 OR #5
#12 #11 AND #4
#13 #12 Refined by: PUBLICATION YEARS: (2020 OR 2019 OR 2018)
Refined by: PUBLICATION YEARS: (2020 OR 2019 OR 2018) AND [excluding] Databases: (MEDLINE) AND [excluding] DOCUMENT TYPES: (MEETING) AND [excluding] DOCUMENT TYPES: (ABSTRACT)

Cochrane Library
Searched on 4 March 2021

ID	Search
#1	MeSH descriptor: [Stroke] explode all trees
#2	MeSH descriptor: [Brain Ischemia] explode all trees
#3	MeSH descriptor: [Brain Infarction] 4 tree(s) exploded
#4	MeSH descriptor: [Cerebral Infarction] 4 tree(s) exploded
#5	(stroke* or ictus or CVA or TIA or apoplex*):ti,ab,kw
#6	((brain* or cerebr* or cerebell* or intracerebral or intracran* or cerebrovasc*) NEAR/3 (accident* or attack* or infarct* or ischaemic* or ischemic*)):ti,ab,kw
#7	((cerebro* NEAR/1 vasc*) or (brain* NEAR/2 vasc*)):ti,ab,kw
#8	#1 or #2 or #3 or #4 #5 or #6 or #7
#9	(((implant* or insert*) NEAR/2 cardiac) NEAR/2 monitor*):ti,ab,kw
#10	((implant* or insert*) NEAR/3 recorder*):ti,ab,kw
#11	(reveal NEAR/2 (linq* or "XT")):ti,ab,kw
#12	biomonitor:ti,ab,kw
#13	(confirm NEAR/2 "RX"):ti,ab,kw
#14	(IMC OR IMCs):ti,ab,kw
#15	#9 OR #10 OR #11 OR #12 OR #13 OR #14
#16	#8 AND #15 with Cochrane Library publication date Between Jan 2018 and Mar 2021

Available from: Martín-Gómez C, Baños-Álvarez E, Isabel-Gómez R, Blasco-Amaro J. Evaluation of the safety, efficacy, effectiveness and cost-effectiveness of implantable Holter for prolonged monitoring in patients with previous stroke: a systematic review. GMS Health Innov Technol. 2023;17:Doc05. DOI: 10.3205/hta000137
