# Supplementary material for: Evaluation of the safety, efficacy, effectiveness and cost-effectiveness of implantable Holter for prolonged monitoring in patients with previous stroke: a systematic review
Source: GMS Health Innov Technol. 2023 Sep 26;17:Doc01. doi: 10.3205/hta000137 (PMC10587481; doi:10.3205/hta000137)
Supplement: Health technology assessment websites which were searched manually [file HINT-17-01-s-002.pdf]

Health technology assessment agency websites that were searched manually

National agencies

- AETSA (Área de Evaluación de Tecnología Sanitaria de Andalucía), AQuAS (Agencia de Calidad y Evaluación Sanitaria de Cataluña)
- IACS (Instituto Aragonés de Ciencias de la Salud)
- AVALIA-T (Agencia de Evaluación de Tecnología Sanitaria de Galicia)
- UETS (Unidad de Evaluación de Tecnología Sanitaria de la Comunidad de Madrid)
- ISCIII (Instituto de Salud Carlos III)
- OSTEBA (Servicio de Evaluación de Tecnologías Sanitarias de Euskadi)
- SECS (Servicio de Evaluación del Servicio Canario de Salud),

International agencies

- KCE (Belgian Health Care Knowledge Centre)
- HAS (French National Authority for Health)
- DIMDI (German Institute for Medical Documentation and Information)
- IQWIG (Institute for Quality and Efficiency in Health Care)
- HIQA (Health Information and Quality Authority)
- Agenas (Agenzia Nazionale per i Servizi Sanitari Regionali)
- ASSR (Regione Emilia Romagna Regional Agency for Health and Social Care)
- ZIN (National Health Care Institute)
- SBU (Swedish Council on Technology Assessment in Health Care)
- TLV (Dental and Pharmaceutical Benefits Agency)
- HIS (Healthcare Improvement Scotland)
- NETSCC (NIHR Evaluation, Trials and Studies Coordinating Centre)
- INHATA (Red Internacional de Agencias de Evaluación de Tecnologías) using the CDR (Centre for Reviews and Dissemination) database
- NICE (National Institute for Health and Care Excellence)
- CADTH (Canadian Agency for Drugs and Technologies in Health)

Available from: Martín-Gómez C, Baños-Álvarez E, Isabel-Gómez R, Blasco-Amaro J. Evaluation of the safety, efficacy, effectiveness and cost-effectiveness of implantable Holter for prolonged monitoring in patients with previous stroke: a systematic review. GMS Health Innov Technol. 2023;17:Doc05. DOI: 10.3205/hta000137
