# Supplementary material for: Evaluation of the safety, efficacy, effectiveness and cost-effectiveness of implantable Holter for prolonged monitoring in patients with previous stroke: a systematic review
Source: GMS Health Innov Technol. 2023 Sep 26;17:Doc01. doi: 10.3205/hta000137 (PMC10587481; doi:10.3205/hta000137)
Supplement: Studies excluded after reading the full text [file HINT-17-01-s-003.pdf]

Studies excluded after Reading the full text

Reason for exclusion
**Different study population**
Reiffel JA, Verma A, Kowey PR, Halperin JL, Gersh BJ, Elkind, MS, et al. Rhythm monitoring strategies in patients at high risk for atrial fibrillation and stroke: a comparative analysis from the REVEAL AF study. Am Heart J. 2020;219:128-36.
Rinciog CI, Sawyer LM, Diamantopoulos A, Elkind MS, Reynolds M, Tsintzos SI, et al. Cost-effectiveness of an insertable cardiac monitor in a high-risk population in the UK. Open Heart. 2020;6: e001037.
Verma A, Wachter R, Kowey PR, Halperin JL, Gersh BJ, Elkind MS, et al. Changes in management following detection of previously unknown atrial fibrillation by an insertable cardiac monitor (from the REVEAL AF study). Am J Cardiol. 2020;124:864-70.
**No comparator, or different comparator**
De Angelis MV, Di Stefano V, Franciotti R, Furia N, Di Girolamo E, Onofrj M et al. Cryptogenic stroke and atrial fibrillation in a real-world population: the role of insertable cardiac monitors. Scientific Reports. 2020;10:1-6.
Diederichsen SZ, Haugan KJ, Brandes A, Graff C, Krieger D, Kronborg C, et al. Incidence and predictors of atrial fibrillation episodes as detected by implantable loop recorder in patients at risk: from the LOOP study. Am Heart J. 2020;219:117-27.
Expósito V, Rodríguez-Entem F, Palacio E, González-Enríquez S, Revilla MA, Vázquez-Higuera JL, et al. Sequential approach for the detection of atrial fibrillation in patients with cryptogenic stroke. REC: CardioClinics. 2019;54:224-30.
Ip J, Jaffe B, Castellani M, Sheikh A, Castellani C, Ip R. Accuracy of arrhythmia detection in implantable cardiac monitors: A prospective randomized clinical trial comparing Reveal LINQ and Confirm Rx. Pacing Clin Electrophysiol. 2020;43:1344-50.
Milstein NS, Musat DL, Allred J, Seiler A, Pimienta J, Oliveros S., et al. Detection of atrial fibrillation using an implantable loop recorder following cryptogenic stroke: implications for post-stroke electrocardiographic monitoring. J Inter Cardiac Electrophysiol. 2020;57:141-7.
Olsen FJ, Christensen LM, Krieger DW, Højberg S, Høst N, Karlsen FM, et al. Relationship between left atrial strain, diastolic dysfunction and subclinical atrial fibrillation in patients with cryptogenic stroke: the SURPRISE echo substudy. Int. J. Card. Imaging. 2020;36:79-89.
Watanabe E, Yamazaki F, Goto T, Asai T, Yamamoto T, Hirooka K, et al. Remote management of pacemaker patients with biennial in-clinic evaluation: continuous home monitoring in the Japanese at-home study: a randomized clinical trial. Circulation: Arrhythmia and Electrophysiology. 2020;13: e007734.
Zakhar J, Blount TJ, Gehi AK, Ferns SJ. Un-LINQed: Spontaneous extrusion of newer generation implantable loop recorders. Indian pacing and electrophysiology journal. 2020;20;:189-92.
**Different intervention**
Kaura A; Sztriha L, Chan FK, Aeron-Thomas J, Gall N, Piechowski-Jozwiak B et al. Early prolonged ambulatory cardiac monitoring in stroke (EPACS): an open-label randomised controlled trial. Eur J Med Res. 2019;24:1-10.
**Different design**
Chorin E, Peterson C, Kogan E, Barbhaiya C, Aizer A, Holmes D, et al. Comparison of the effect of atrial fibrillation detection algorithms in patients with cryptogenic stroke using implantable loop recorders. Am J Cardiol. 2020:129:25-9.
Oliveira TDS, Freitas JPP. Registadores de Eventos Implantados no Diagnóstico de Síncope: Role of Implantable Loop Recorders. Medicina Interna. 2019;26:126-34.
Triantafyllou S; Katsanos AH; Dilaveris P; Giannopoulos G; Kossyvakis C; Adreanides E. et al. Implantable cardiac monitoring in the secondary prevention of cryptogenic stroke. Ann Neurol. 2020;88:946-55.
**Study type and date of publication**
Bernstein RA, Kamel H, Granger CB, Kowal RC, Ziegler PD, Schwamm, LH. Stroke of Known Cause and Underlying Atrial Fibrillation (STROKE-AF) randomized trial: design and rationale. Am Heart J. 2017;190:19-24.
Buck, BH. Post-Embolic Rhythm Detection with Implantable Versus External Monitoring (per diem). NCT02428140. URL:https://clinicaltrials.gov/ct2/show/NCT02428140

Edwards SJ, Jhita T, Cain P, Wakefield V, Kew K, Marceniuk G. Cost Effectiveness Analysis of Implantable Cardiac Monitors To Detect Atrial Fibrillation After Cryptogenic Stroke. 2019;22 (suppl 3):S673.
Kaura A, Sztriha L, Chan FK, Aeron-Thomas J, Gall N, Piechowski-Jozwiak B et al. Early prolonged ambulatory cardiac monitoring in stroke (epacs): randomised clinical trial using patch-based cardiac monitoring. Póster presentado en ESC Congress and World Congress of Cardiology; 2019 Ago 31- Sept 4; Paris, Francia.
Reinke F, Bettin M, Ross LS, Kochhäuser S, Kleffner I, Ritter M, et al. Refinement of detecting atrial fibrillation in stroke patients: results from the TRACK-AF Study. Eur J Neurol. 2017;25:631-6.
Romanow AB, Pokushalov E, Ponomarev D, Shabanov V, Losik D, Elesin D, et al. Does atrial fibrillation burden predict clinical outcome after atrial fibrillation interventional therapy: pooled analysis of two randomized studies using continuous rhythm monitoring. Póster presentado en Heart Rhythm 40th Scientific Sessions; 2019 May 8-11; San Francisco, Estados Unidos.
Wachter R. Intensive Rhythm Monitoring to Decrease Ischemic Stroke and Systemic Embolism - the Find-AF 2 Study. NCT04371055. URL: https://clinicaltrials.gov/ct2/show/NCT04371055
Yokokawa M, Jaffe B, Ip R, Azad R, Castellani M, Ip J. Efficiency and accuracy of arrhythmia detection using implantable cardiac monitor: A prospective multicenter randomized clinical trial comparing Reveal LINQ and Confirm Rx. Póster presentado en ESC Congress and World Congress of Cardiology; 2019 Ago 31- Sept 4; Paris, Francia. DOI: 10.1093/eurheartj/ehz746.0591
Yokokawa M, B Jaffe, R Ip, C Castellani, J Ip. Accuracy of arrhythmia detection using confirm Rx implantable cardiac monitor: a prospective multicenter randomized clinical trial. Póster presentado en: APHRS 2019. 12th Asia Pacific Heart Rhythm Society Scientific Session; 2019 Oct 4-27; Bangkok, Thailand. DOI: doi.org/10.1002/joa3.12273
M Yokokawa, R Ip, A Sheikh, M CastellanI, J Ip. Accuracy of implantable cardiac monitor in diagnosing cardiac arrhythmias: A prospective randomized clinical trial comparing reveal linq trurhythmtm versus confirm RX sharpsensetm. Póster presentado en ACC.20. World Congress of Cardiology; 2020 Mar 28-30; Chicago, Estados Unidos.

Available from: Martín-Gómez C, Baños-Álvarez E, Isabel-Gómez R, Blasco-Amaro J. Evaluation of the safety, efficacy, effectiveness and cost-effectiveness of implantable Holter for polonged monitoring in patients with previous stroke: a systematic review. GMS Health Innov Technol. 2023;17:Doc05. DOI: 10.3205/hta000137
