# Supplementary material for: Evaluation of the safety, efficacy, effectiveness and cost-effectiveness of implantable Holter for prolonged monitoring in patients with previous stroke: a systematic review
Source: GMS Health Innov Technol. 2023 Sep 26;17:Doc01. doi: 10.3205/hta000137 (PMC10587481; doi:10.3205/hta000137)
Supplement: Methodological quality of the included studies [file HINT-17-01-s-004.pdf]

Methodological quality of the included studies

Methodological quality assessment tool	AMSTAR-2																Overall assessment
	Items																
**Author, year**	1	2	3	4	5	6	7	8	9	10	11	12	13	14	15	16	
Edwards et al., 2020	Y	Y	Y	Y	Y	Y	Y	Y	N	Y	NM	NM	Y	Y	NM	Y	Low
Chew et al., 2019	Y	N	N	PY	Y	Y	Y	Y	Y	Y	NM	NM	N	Y	NM	Y	Low

Methodological quality assessment tool	AGREE-II						Overall assessment
	Domains						
**Author, year**	1	2	3	4	5	6	
NICE, 2020	100%	78%	81%	100%	38%	100%	82.83%[a]

Methodological quality assessment tool	CHEC-list																			Overall assessment
	Items																			
**Author, year.**	**1**	**2**	**3**	**4**	**5**	**6**	**7**	**8**	**9**	**10**	**11**	**12**	**13**	**14**	**15**	**16**	**17**	**18**	**19**	
Maervoet, et al., 2019	Y	Y	N	Y	Y	Y	Y	N	Y	Y	Y	Y	Y	Y	Y	Y	Y	Y	N	Moderate

NM= No meta-analysis conducted; Y= Yes; N= No; PY = Partial yes; [a] Values above 70% represents adequate quality.

Available from: Martín-Gómez C, Baños-Álvarez E, Isabel-Gómez R, Blasco-Amaro J. Evaluation of the safety, efficacy, effectiveness and cost-effectiveness of implantable Holter for polonged monitoring in patients with previous stroke: a systematic review. GMS Health Innov Technol. 2023;17:Doc05. DOI: 10.3205/hta000137
